# Determinants of urban sprawl in European cities

**DOI:** 10.1177/0042098015577773

**Published:** 2015-07

**Authors:** Walid Oueslati, Seraphim Alvanides, Guy Garrod

**Affiliations:** Agrocampus Ouest, France; Northumbria University, UK; University of Newcastle, UK

**Keywords:** European cities, monocentric city model, spatial scale, urban fragmentation, urban scattering, urban sprawl

## Abstract

This paper provides empirical evidence that helps to answer several key questions relating to the extent of urban sprawl in Europe. Building on the monocentric city model, this study uses existing data sources to derive a set of panel data for 282 European cities at three time points (1990, 2000 and 2006). Two indices of urban sprawl are calculated that, respectively, reflect changes in artificial area and the levels of urban fragmentation for each city. These are supplemented by a set of data on various economic and geographical variables that might explain the variation of the two indices. Using a Hausman-Taylor estimator and random regressors to control for the possible correlation between explanatory variables and unobservable city-level effects, we find that the fundamental conclusions of the standard monocentric model are valid in the European context for both indices. Although the variables generated by the monocentric model explain a large part of the variation of artificial area, their explanatory power for modelling the fragmentation index is relatively low.

## Introduction

Europe has one of the world’s highest densities of urban settlement, with over 75% of the population living in urban areas. Despite Europe’s relatively low rate of population growth, there continues to be an uneven expansion of urban areas across the continent. The size of many European cities is increasing at a much faster rate than their populations. This trend towards reduced population densities began in the early 1970s, most prominently in medium-sized European cities. There is no sign that this trend is slowing down and, as a result, the demand for land around cities is becoming a critical issue in many areas (EEA (European Environment Agency), 2006).

The phenomenon of increasingly large urban areas taking up a greater proportion of the available land area is often termed urban sprawl. Various studies such as Czech et al. (2000), Johnson (2001) and Robinson et al. (2005) have documented the negative environmental impacts that can be linked to urban sprawl, while other studies (e.g. Hasse and Lathrop, 2003) have discussed the increased social costs associated with the provision of public infrastructure as cities increase in size. Such impacts can be expected to have a negative effect on the quality of life of people living in European cities. For this reason, it is essential to gain a better understanding of urban sprawl and to gain some insights into its causes. This paper, therefore, sets out to explore the determinants of urban sprawl in European cities and to compare its findings with those of the existing literature on this topic.

The literature on urban sprawl incorporates the work of economists, geographers and planners. Surveys of important issues underlying this research can be found in, among others, Anas et al. (1998), Brueckner (2000), Nechyba and Walsh (2004), Couch et al. (2007) and Anas and Pines (2008). Although there is some debate over the precise definition of urban sprawl, a general consensus seems to be emerging that characterises urban sprawl as a multidimensional phenomenon, typified by an unplanned and uneven pattern of urban development that is driven by a multitude of processes and which leads to the inefficient utilisation of land resources. Urban sprawl is observed globally, though its characteristics and impacts vary. While early research in this area tended to focus on North American, several recent studies have discussed the acceleration of urban sprawl across Europe (e.g. Couch et al., 2007; Christiansen and Loftsgarden, 2011; EEA, 2006). Although differences in the nature and pattern of sprawl have been observed between Europe and North America, there are also intra-European variations in urban sprawl reflecting the former’s greater diversity in geography, land-use policy, economic conditions and urban culture.

Despite the increasing interest in urban sprawl in Europe, relatively few empirical studies have been undertaken at the continental scale. The heterogeneity of the European urban context and limitations with the availability of data are probably the main reasons for this lack of interest. That is not to say that there has not been an effort to study the process of urban sprawl in Europe. Various studies, including Batty et al. (2003), Phelps and Parsons (2003), Holden and Norland (2005), Couch et al. (2007), Travisi et al. (2010) and Pirotte and Madre (2011), have focused on urban sprawl within particular regions or cities. However, to the best of our knowledge, only Patacchini and Zenou (2009) and Arribas-Bel et al. (2011) have attempted a general overview of the phenomenon for Europe as a whole.

Patacchini and Zenou (2009) sought to contrast urban patterns in Europe and the USA, using data on a sample of European Cities.^1^ They noted the lack of a standard definition of the city or metropolitan area in Europe and highlighted the difficulties inherent in attempting a systematic cross-national comparison of European cities owing to the limited availability of data. Despite these limitations, their study provided some evidence on the extent of urban sprawl in cities in the European Union. However, their study did not address the measurement of urbanised areas per se and instead concentrated on identifying factors that influence population density. In their study, Arribas-Bel et al. (2011) used spatial data, derived from the European Corine Land Cover database to consider the issue of urban sprawl from a multidimensional viewpoint. Using six dimensions to define the concept (i.e. connectivity, decentralisation, density, scattering, availability of open space and land-use mix), they developed various indices of sprawl that were then calculated for a sample of 209 European cities for the year 2000. Even though their study offered a new methodological approach using rich data sets to measure urban sprawl, it did not explicitly address the determinants of the phenomenon.

The main objective of our paper is to explore the relationship between urban sprawl and a set of variables that urban economic theory and the empirical literature suggest may be correlated with the phenomenon. Thus, rather than attempting to determine the causal factors behind urban sprawl we adopt a heuristic approach and seek to test competing hypotheses on the impact of various determinants. To this end we identify and gather existing data that can be used to identify the key determinants of urban sprawl across a large sample of European cities. Analysis is based on the well-known monocentric city model, which identifies population, income, commuting costs and the value of land as essential drivers of sprawl. In addition to these economic variables, the impacts of other geographical, socio-cultural and climatic factors, suggested by the literature, are also considered.

Our study makes two main contributions to the literature on urban sprawl. The first concerns the measurement of sprawl and provides some observations about the data that are available for this purpose in Europe. Two complementary indices of sprawl are used in this paper, the first reflecting a change in spatial scale and the second the degree of fragmentation that is observed when large urban areas grow. Here, the term fragmentation is used to describe an aspect of urban morphology reflecting the spatial pattern of urban development, and in particular whether such development is compact or scattered in various fragments separated by open spaces. The extent of sprawl increases as development becomes more dispersed and further non-contiguous fragments are added to the urban area. The processes underlying the scattered development of urban areas have been discussed by Burchfield et al. (2006) among others.

By considering these two indices, we seek to ascertain whether or not the factors associated with the expansion of urban areas are also linked to an increase in fragmentation, with the city becoming less compact as the distances between adjacent patches of urban land increase. Both indices are calculated using Corine Land Cover data sets for three reference years (1990, 2000 and 2006). Several data sources are used to build a wide-ranging and consistent set of explanatory variables for a sample of 282 European cities. To our knowledge this is the first time that a study of this magnitude and scope has been conducted in the European context.

The second important contribution of this study is related to the econometric techniques used in the estimation of the indices. Unlike previous studies, a comprehensive analysis of panel data is conducted to account for unobservable individual heterogeneity and to determine the best estimation method for each index. Several tests were used to choose between alternative panel data estimators. Specifically, a modified random effects-type model (the Hausman–Taylor model) is used, which allows us to control for endogeneity bias while, simultaneously, identifying the estimates for the time-invariant regressor.

## Background

The fundamental theory in urban economics relevant to urban expansion is the monocentric city model (Alonso, 1964; Mills, 1981; Muth, 1961; Wheaton, 1974). Within this model it is assumed that all employment in the city takes place within a single Central Business District (CBD). The pattern of urban development is then shaped by the trade-off between affordable housing further away from the CBD and the associated commuting costs. Thus, to offset higher commuting costs, housing prices decline with distance away from the CBD.

There are four main predictions of the monocentric city model. First an increase in the urban population should increase the distance to the edge of the city and raise the population density, since more people need to be housed. Second, an increase in income increases housing demand and leads to an extended city with a lower population density. Third, an increase in commuting cost lowers disposable income at all locations, reducing housing demand and leading to a more compact city with higher population density. Fourth, increasing the agricultural land rent raises the opportunity cost of urban land and makes the city smaller and denser.

This basic version of the monocentric city model cannot explain the scattered development observed in some cities, where some parcels of land are left undeveloped while others, further away, are built up. One way of accounting for scattered development is to assign an amenity value to public open space, so that individuals may be willing to incur the additional commuting costs associated with living further away from the CBD in order to have open space near their home (Newburn and Berck, 2011; Tajibaeva et al., 2008; Turner, 2005; Wu, 2006; Wu and Plantinga, 2003). Similarly, Cavailhès et al. (2004) and Coisnon et al. (2014) showed that the spatial heterogeneity of agricultural amenities can also lead to leapfrog development within a suburban area.

Several studies have tested the empirical validity of the monocentric city model. In independent studies of the US context, Brueckner and Fansler (1983), McGrath (2005) and Song and Zenou (2006), all found that income, population and agricultural rent were statistically significant determinants of urban land area. However, the coefficients of the variables relating to commuting costs were ambiguous and varied depending on the proxies used to measure them.

In addition to the key variables of the monocentric city model, a study by Burchfield et al. (2006) included additional environmental and spatial variables to account for differences between cities. In their study, sprawl is measured as the amount of undeveloped land surrounding an average urban dwelling. This approach captures the extent to which urban development is scattered across undeveloped land. The study concluded that sprawl in the USA between 1976 and 1992 was positively related to groundwater availability, temperate climate, rugged terrain, decentralised employment, early public transport infrastructure, uncertainty about metropolitan growth and the low impact of public service financing on local taxpayers.

In a developing country context, Deng et al. (2008) and Shanzi et al. (2009) investigated the determinants of the spatial scale of Chinese cities using a consolidated monocentric city model. Their results demonstrate the crucial role that income growth has played in China’s urban expansion. Similarly, while Deng et al. (2008) found that industrialisation and the rise of the service sector both appear to have influenced the growth of urban development, they conclude that the role of these factors was relatively minor compared with the direct effect of economic growth.

These studies confirm that the monocentric city model is empirically robust. The economic variables identified by this literature explain the majority of variation in the sizes of cities in different contexts. Moreover, many other geographical variables have also been found to play an important role in explaining urban expansion.

It should also be noted that some models have included variables that measure the ethnic composition of the population (e.g. Selod and Zenou, 2006) and crime rates (e.g. Freeman et al., 1996). In the American context, it was established that increases in the percentage of ethnic minority populations within cities and rising city centre crime rates both led to a growth in urban sprawl. The latter has been explained by the desire of many residents to improve their personal security by moving further away from the central area of the city. In a European context, Patacchini and Zenou (2009) confirmed the positive impact of higher crime rates on sprawl, but observed the opposite effect for the impact of ethnic minority populations.

Our paper contributes to this literature by explicitly considering the multidimensional nature of sprawl. Although there is evidence that urban sprawl is a multidimensional issue that should be measured in a particular way, each of the previous empirical studies examines only a single dimension of sprawl, i.e. either the urbanised area or the population density. In contrast, Chin (2002) identified three main dimensions of urban sprawl, respectively based around urban spatial scale, population density decline and scattered urbanisation. These dimensions provide the rationale for the indicators used in our study. Based on this approach, we revisit the key aspects of the monocentric city model within the European context.

## Data

Based on the theoretical and empirical literature, we seek to explain differences in urban sprawl in Europe across space and time. The approach taken here is mainly based on the monocentric city framework and conceptually the empirical model is given by:

(1) $$\begin{array}{cc} & \mathrm{Sprawl}\;\mathrm{index}=f(\mathrm{income},\mathrm{population},\\ & \mathrm{agricultural}\;\mathrm{land}\;\mathrm{value},\mathrm{transportation}\\ & \;\mathrm{costs},\mathrm{other}\;\mathrm{socio}-\mathrm{economic},\mathrm{climatic}\\ & \mathrm{and}\;\mathrm{geographic}\;\mathrm{variables}) \end{array}$$

This study considers two indices of sprawl that reflect the spatial scale of cities and urban morphology. By considering these indices, we examine the extent to which the determinants of urban expansion are linked to the fragmentation of urban areas. As independent variables, both indices will be estimated using the same explanatory variables. In this section we specify the sources and extent of the data used in this study before discussing the methodological features of sprawl measurements and the choice of explanatory variables.

### Data on urbanisation and sprawl measures

We focus on a sample of European cities obtained by combining various existing data sources. Our starting point was the complete set of 320 cities used in the Urban Audit (UA) database.^2^ Here, all cities are defined at three scales: the Core city, which encompasses the administrative boundaries of the city; the Large Urban Zone (LUZ), which is an approximation of the functional urban region centred around the Core city; and the Sub-City District, which is a subdivision of the LUZ (Eurostat, 2004). We concentrate on LUZs, because sprawl is observed around the fringes of cities from where it spreads out across the whole urban region. Therefore, the boundaries of each LUZ define the spatial units upon which this study is based.

**Figure 1.**
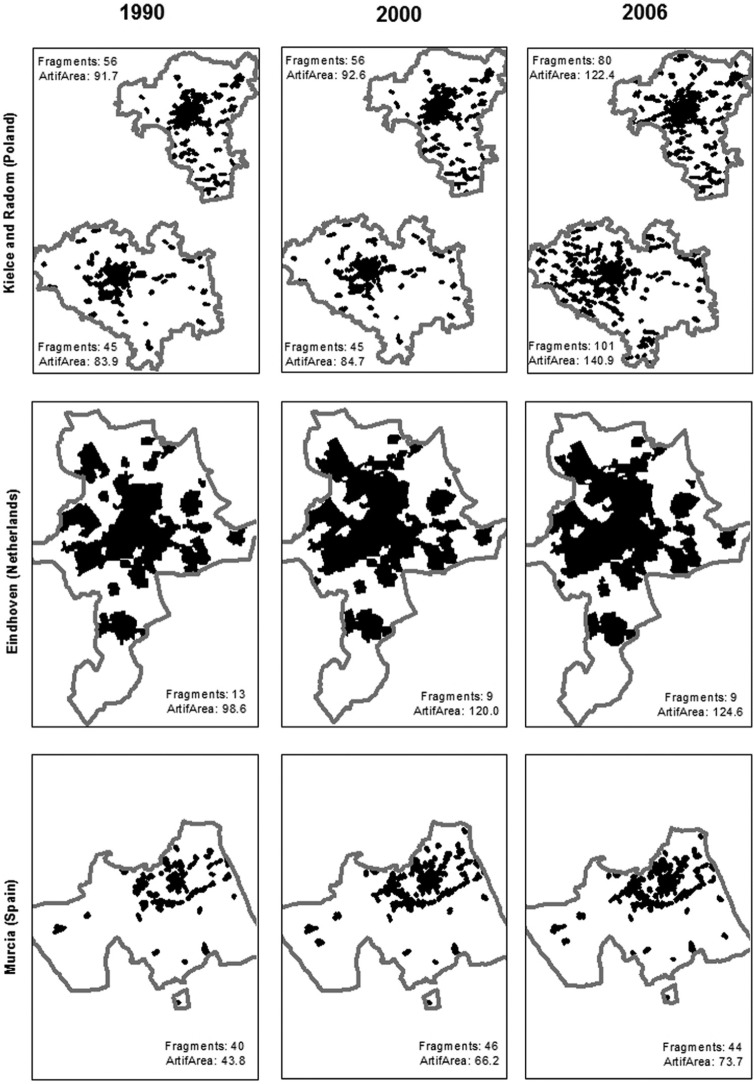
UMZ boundaries (in grey) and artificial urban areas (in black) for selected cities.

UA provides rather limited information on land use, with poor coverage for many cities. As an alternative to this data set, we use data on Urban Morphological Zones (UMZ), compiled by the EEA, which contains spatial information for three years (1990, 2000 and 2006).^3^ Derived from Corine Land Cover, UMZ data covers the whole EU-27 at a 200 m resolution for those urban areas considered to contribute to urban tissue and function (Guérois et al., 2012). Geospatial data on land use for each city is obtained by superimposing the LUZ boundaries onto the UMZ spatial data, using a Geographical Information System (GIS). To illustrate this process and the nature of the spatial data, Figure 1 provides maps documenting the changes in the urban (artificial) area for four selected cities: Kielce and Radom (Poland), Eindhoven (Netherlands) and Murcia (Spain), over the three snapshot years for which the UMZ data were available. While the external boundary (shown in grey) of each LUZ remains stationary over time, the fragments of urban land (i.e. the artificial area) within the boundaries (represented by the black patches) vary in both size and quantity. The cities in Figure 1 were chosen to illustrate different urban dynamics. For Kielce and Radom, both the number of fragments and the total artificial area increased significantly in a relatively short period of time (between 2000 and 2006). In Eindhoven the total artificial area increased between 1990 and 2006, even though the number of fragments decreased. Finally, during the same period, Murcia experienced major urban development (as evidenced by the overall increase in artificial area), but the number of fragments remained relatively constant.

**Figure 2.**
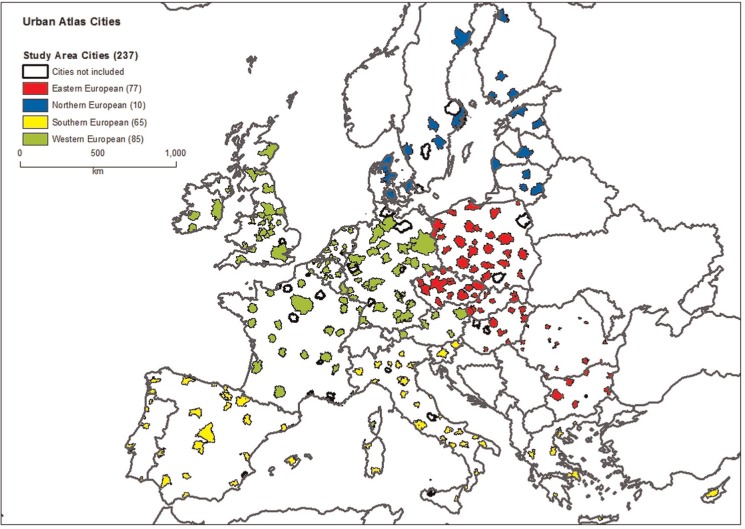
Study area with Urban Atlas Cities for supra-national regions.

While the UA database covers 320 cities, the publicly available UMZ data does not include information for the UK, Cyprus and Finland for 1990 and for Greece and Cyprus for 2006. Taking this into account, the sample used in this study comprises the 237 LUZs where, for 1990, 2000 and 2006, complete information is available on artificial area and the number of urban fragments. Figure 2 shows the extent of the sample and the individual cities are identified by four colours, depending on their supra-national region group.^4^

Two indices of urban sprawl are constructed. The first index aims to measure the spatial scale of each city by considering the total artificial area in square kilometres (ArtifArea) as a proxy for all urbanised land in each LUZ. These areas were obtained directly from the spatial UMZ data according to Corine Land Cover nomenclature. This simple measure reflects the evolution of urban land cover in a given area without any prejudgment on internal composition or urban morphology, i.e. the scattered nature of the urban area.

The second index reflects urban morphology, and the spatial patterns of residential land development, in particular, whether residential development is scattered or compact. A simple scattering index is adopted, measuring the degree to which urban development is spread across land in different fragments. We use the following expression:

(2) $$\mathrm{Scatt}=\frac{\mathrm{Frag}}{\mathrm{ArtifArea}}$$

where *Frag* represents the number of urban fragments (i.e. individual patches) within a specific LUZ. This index reflects how scattered urban development is across the whole urban region and sprawl is identified as a higher number of different fragments. Since larger urban areas would be expected to have more fragments than smaller ones, this size effect is corrected by dividing *Frag* by the artificial area within each LUZ.

### Data on explanatory variables

The UA database provides a wide range of variables, including those commonly used in the monocentric city model (e.g. urban area, population, revenue, transport). However, data are missing for some cities, which makes the use of some variables unfeasible. Therefore, the UA data are supplemented by data obtained from the European Observation Network, Territorial Development and Cohesion (ESPON).^5^ When combined, these data sources provide a set of explanatory variables covering a broad sample of European cities.

The ESPON database provides comprehensive data for each LUZ on Gross Domestic Product (GDP) adjusted for Purchasing Power Standards^6^ and total population (POP).^7^ GDP per capita (GDPcap) is used as a proxy for income. All of these variables are defined for the three reference years (1990, 2000 and 2006) for 282 cities across Europe. However, no direct measures of transport costs or agricultural land rents exist for the whole of Europe over the relevant time periods. Similarly, there are no European data sets relating to agricultural land markets or transport costs at the city level. Based on the empirical studies cited earlier, proxies were identified that provide adequate measurements of these variables. First, to account for agricultural land rent, the ratio of agricultural added value to the area of agricultural land (Agriprox) was calculated. Data on agricultural added value were available from ESPON, and the agricultural land area for each LUZ was calculated. The rationale for including this proxy is that this ratio could explain different levels of agricultural productivity. Normally, higher agricultural productivity should be capitalised into land rent. Similarly, highway density (Highway) data from the Eurostat regional data set was used as a proxy for transport costs. The assumption here, is that investments in highways make travelling faster and more convenient, which reduces the time and the costs of commuting.

Following Burchfield et al. (2006) and Deng et al. (2008), a set of climatic and environmental data was collected from the UA database. The former includes the number of days of rain per year (Rain) and the average temperature of the warmest months of the year (Temperature). The latter includes the annual average concentration of NO_2_ (*NO2*) as a good indicator of air pollution. A terrain variable, *median city centre altitude above sea level* (MedAlt), is also included. This variable is a partial indicator for the ruggedness of the terrain in the LUZ, as this may have an impact on the potential for urban growth.

In addition to the economic and geographical variables of interest, other social and cultural variables are considered. First, data on recorded crime (Crime) from the UA is used to account for the security situation in the central city. As mentioned previously, Patacchini and Zenou (2009) found that higher crime rates increase sprawl. Second, the number of cinema seats (CineSeats) is included as a proxy for the cultural attractiveness of the central city. A vibrant central city would be expected to discourage decentralisation, thus reducing sprawl and resulting in more compact urban areas. Despite some of these variables having missing data for certain cities, they were used to estimate the differentiating factors between different LUZs in the sample. Table 1 provides a statistical summary of the panel data used in this study.

**Table 1.** Statistical summary of explanatory variables.

Variables *unit /(source)*^c^	Obs.^a^	Missing obs.^b^	Mean	Min		Max	St. dev.	
**ArtifArea***km^2^(UMZ)*	801	45	211.41	9.64	2876.50			293.54
***Scatt****fragmenst/km (UMZ)*	801	45	0.472	0.017	1.438			0.275
**POP**^(a)^*1000 inhabitants (ES)*	846	0	939.8	26.7	12,961			1255.7
**GDPcap**^a^*Euros (ES)*	846	0	19,935.6	1152	149,681			12,288.2
**Agriprox***Euros/ha (ES and U)*	240	42	5761.9	36.2	90,364.2			10,415.2
**Highway***km/km^2^(ER)*	282	0	28.6	0.1	289.0			36.4
**Crime** per *1000 inhabitants (UA)*	228	54	79.1	0.9	233.0			45.4
**Rain***Number of days of rain per year (UA)*	282	0	157.3	32.0	266.0			49.6
**Temperature***°C (UA)*	282	0	21.2	14.6	35.5			4.0
**AccessAir***EU=100(UA)*	248	34	94.6	26.0	187.0			34.4
**NO_2_***Annual average concentration (UA)*	210	72	27.6	8.7	64.8			10.3
**CineSeats***per1000 inhabitants (UA)*	250	32	17.3	0.8	51.9			9.8
**MedAlt***m (UA)*	282	0	132.2	2	746			142.5
**W** Dummy (= 1 if western European city, 0 otherwise)^d^	282	0	0.387	0	1			0.487
**S** Dummy (= 1 if southern European city, 0 otherwise)^d^	282	0	0.266	0	1			0.442
**E** Dummy (= 1 if eastern European city, 0 otherwise)^d^	282	0	0.294	0	1			0.456

*Notes*: ^a^The sample consisted of 282 cities observed in 1990, 2000 and 2006.

b Missing data includes cities in the UK and Greece for 1990, and Cyprus, Finland, Greece, Sweden for 2006.

c Data sources: ER: Eurosatat Regional data; ES: ESPON; U: UMZ and UA.

d North is taken as the reference.

### Variations in urban sprawl across Europe

Preliminary analysis of the data shows that on average, over the period 1990–2006, the urbanised area increased by 18.4%, while population density fell by 9.43% and the Scattering index decreased by 9.07% (Table 2). In general, European cities became larger, less dense and more compact over this period. Obviously these averages conceal a wide variation across countries and regions. To observe the evolution of sprawl indices at the regional level, the sample is divided into four supra-national regions as shown in Figure 2. Table 2 shows that the Southern European cities achieved the highest urban growth (32.02 %), but with less fragmentation of urban areas (−13.98%). Despite low growth in urban areas, the Eastern cities are denser and more scattered. The western European cities experienced high urban growth (15.29%), small decrease in density (−3.8%) and a decrease in scattering close to the sample mean. Northern European cities showed low urban growth (7.98%) but a sharp decline in density (−11.91%) and scattering (−8.08%).

**Table 2.** Growth rates of sprawl indices, population and GDP between 1990 and 2006 according to different supra-national region groups.

Sprawl indices in growth rate *1990–2006 (per cent)*	Obs.^a^	*ArtifArea*	Scatt	Density
*All cities*	237	18.40	− 9.07	− 9.43
*Southern European cities*	65	32.02	−13.98	−14.53
*Western European cities*	85	15.29	−9.62	−3.80
*Eastern European cities*	77	11.68	−4.36	−11.01
*Northern European cities*	10	7.98	−8.08	−11.91

*Note*: ^a^Only includes cities for which urbanisation data for 1990 and 2006 are available.

In summary, sprawl shows different trends depending on the index used to measure it and the region within which cities are located. Southern cities show the fastest growth of urbanisation and the highest decrease in density, but their morphology tends to be more compact. Despite their relatively low levels of urban growth, northern cities experienced a relatively large decline in both density and scattering. From this it can be deduced that rapid urbanisation is not necessarily accompanied by a decrease in density. Also, that urban areas within cities where density is declining do not necessarily become more scattered.

To illustrate the interdependence between these three indices of urban sprawl and the time-varying explanatory variables (Population and GDP per capita), the sample of LUZs is divided into two groups depending on the rate of growth of each index. The first group corresponds to the bottom quartile (relatively slow growth) and the second group corresponds to the top quartile (relatively high growth). Table 3 summarises changes across these groups. Inspection of Table 3 shows that GDP per capita growth is lower for cities where growth is relatively slow, compared with those growing at a much faster rate. However, GDP per capita growth is inversely related to density and the growth of scattering. It should also be noted that population growth is lower for cities having a slow growth of urbanisation and density, but is higher for cities with a slow growth of scattering.

**Table 3.** The growth rate of sprawl indices between 1990 and 2006 according to the change of population and GDP per capita.

Items	Population growth (per cent)	GDPcap growth (percent)
Relatively slow *ArtifArea* growth^a^	−0.50	10.26
Relatively high *ArtifArea* growth^b^	70.92	77.11
Relatively slow density growth^a^	2.05	77.55
Relatively high density growth^b^	8.77	56.06
Relatively slow scatt growth^a^	7.44	78.44
Relatively high scatt growth^b^	1.20	68.16

*Notes*: ^a^Relatively slow growth is associated with the cities that are in the lowest quartile.

b Relatively high growth is associated with cities that are in the highest quartile.

All changes in urban areas and density are in the directions predicted by the monocentric city model. Furthermore, the growth of population and GDP per capita are negatively correlated with the evolution of scattering.

## Empirical model and regression results

### Estimation strategy

Panel data analysis is adopted to deal with observations from multiple cities over three periods. Given the variables discussed above, the estimating equation of sprawl indices is given by:

(3) $$\begin{array}{c} \log({\mathrm{SI}}_{\mathrm{it}})=\alpha_{i}+\beta \log\left({\mathrm{POP}}_{\mathrm{it}}\right)\\ +\mu \log({\mathrm{GDPcap}}_{\mathrm{it}})+\gamma_{i}D_{i}+\varepsilon_{\mathrm{it}} \end{array}$$

where *i* and *t* stand for cities and time periods, respectively. The dependant variable ${\mathrm{SI}}_{\mathrm{it}}$ represents urban sprawl indices (*ArtifArea* or *Scatt*).^8^ There are two time-varying regressors, Population (*POP*) and GDP per capita (*GDPcap*). $D_{i}$ is a vector of time-invariant variables. $\alpha_{i}$ is specified as random or fixed effects. $\varepsilon_{\mathrm{it}}$ is the error term.^9^

Equation (3) may be estimated using ordinary least squares (OLS) regression, pooling observations across cities and over time. However, OLS does not take the panel nature of the data into account and could therefore result in invalid inferences being made from the data (Baltagi, 2005).^10^ Rather than use OLS in this case, it is more appropriate to use random and fixed-effects models both of which are commonly applied to panel data. Both models take account of unobservable individual heterogeneity and the distinction between them is whether or not the individual-specific time-invariant effects are correlated with the regressors. The fixed-effects model offers consistent estimators but does not allow us to estimate time-invariant variables since it is based on the within operator (i.e. it subtracts from variables their mean over time, so time-invariant variables have a mean equal to their value and the within estimator leads to a null value of the within transformation of these variables). The random- effects model increases the efficiency of estimations but imposes a strong assumption that individual effects are not correlated with explanatory variables.

Furthermore, in order to improve on some of the shortcomings of these two models, the Hausman-Taylor instrumental variable estimator can also be applied here (Hausman and Taylor, 1981). The Hausman-Taylor model combines the fixed and random-effects models to deal with the null correlation between the specific effects and the covariates by allowing some variables to be considered as endogenous, i.e. correlated with individual effects. The variance matrix of the composite errors maintains the random structure but the variables suspected of being correlated with the individual effects are instrumented by their within transformation (Wooldridge, 2002).

The model selection process follows Baltagi et al. (2003) in using the Hausman test to select between alternative panel data estimators (Hausman, 1978). First, we perform a Hausman test comparing the fixed and random-effects estimators. If the null hypothesis of no systematic differences is not rejected, the random-effects model is preferred since it yields the most efficient estimator under the assumption of no correlation between the explanatory variables and the errors. However, if the Hausman test between fixed and random effects is rejected, then a second Hausman test is performed comparing the Hausman-Taylor estimator and the fixed effects estimator. Failure to reject this second Hausman test implies the use of the more efficient Hausman-Taylor estimator, while rejection implies the use of the fixed model.^11^

The Hausman and Taylor method can be represented in its most general form as follows:

(4) $$\begin{array}{cc} Y_{\mathrm{it}}= & X_{1.\mathrm{it}}\beta_{1}+X_{2.\mathrm{it}}\beta_{2}+Z_{1.i}\gamma_{1}\\ & +Z_{2.i}\gamma_{2}+v_{i}+\varepsilon_{\mathrm{it}} \end{array}$$

where $X_{1.\mathrm{it}}$ and $X_{2.\mathrm{it}}$ are time-varying variables, whereas $Z_{1.i}$ and $Z_{2.i}$ are individual time-invariant regressors. $v_{i}$ is iid(0. $\sigma_{v}^{2})\;\mathrm{and}\;\varepsilon_{\mathrm{it}}$ is idd(0. $\sigma_{\varepsilon }^{2})$ and both are independent of each other. The $X_{1}$ and $Z_{1}$ are assumed to be exogenous and not correlated with $v_{i}$ and $\varepsilon_{\mathrm{it}}$. while the $X_{2}$ and $Z_{2}$ are endogenous because of their correlation with $v_{i}$ but not with $\varepsilon_{\mathrm{it}}$. Thus, the endogeneity arises from the potential correlation with individual fixed effects. Hausman and Taylor (1981) suggest an instrumental variables estimator which premultiplies expression (4) by $1/\sqrt{\Delta }$ (where Δ is the variance-covariance term of the error component $v_{i}$+$\varepsilon_{\mathrm{it}}$) and then performs two-stage least squares (2SLS) using as instruments [Q, X_1_, Z_1_], where Q is the within transformation matrix with $X_{\mathrm{it}}^{*}=\mathrm{QX}$ having a typical element $X_{\mathrm{it}}^{*}=X_{\mathrm{it}}-{\bar{X}}_{i}$ and ${\bar{X}}_{i}$ the individual mean. Thus 2SLS is performed with [$X^{*}.{\bar{X}}_{1}\;.Z_{1}$] as the set of instruments (Baltagi et al., 2003). If the model is identified, in the sense that there are at least as many time-varying exogenous regressors $X_{1}$ as there are individual time-invariant endogenous regressors $Z_{2}$, then this Hausman-Taylor estimator is more efficient than the fixed-effects estimator. How should the endogenous and exogenous variables be defined? The Hausman-Taylor estimator should produce estimations close to the fixed-effect estimator for time-varying variables. Thus, a Hausman test between the fixed-effects model and the Hausman-Taylor model allows the best specification to be chosen.

### Results

A Hausman test is used to discriminate between fixed and random-effects approaches. Under the null hypothesis of the Hausman test, the estimators from the random-effects model are not systematically different from those from the fixed-effects model. If the null hypothesis cannot be rejected (probability of the test higher than 5%), we consider the estimators from the random-effects model to be consistent. Otherwise, if the null hypothesis is rejected (probability lower than 5%) only the fixed-effects model is consistent and unbiased. In the case of this model, Hausman test results show that the random-effects hypothesis is rejected in favour of the fixed-effects estimator when ArtifArea index is the dependent variable. However, when the Scatt index is considered as the dependent variable, the random-effect regressor is consistent. The results of the Hausman test are reported in the bottom of Tables 4 and 5.^12^

**Table 4.** Estimation of the determinants of ArtifArea index (Hausman-Taylor).

	Dependent variable : Ln(ArtifArea)			
	(1)	(2)	(3)	(4)
*Constant*	3.788 (6.76)**	2.752 (6.45)**	2.817 (1.066)	2.095 (0.83)
*Ln(POP)*	0.288 (6.08)**	0.329 (6.98)**	0.170 (2.96)**	0.185 (3.24)**
*Ln(GDPcap)*	0.168 (5.38)**	0.246 (17.83)**	0.210 (4.71)**	0.281 (15.14)**
*Ln(Agriprox)*	−0.270 (7.80)**	−0.265 (2.89)**	−0.279 (5.80)**	−0.277 (5.96)**
*Ln(Highway)*	0.103 (4.27)**	0.095 (4.20)**	0.084 (2.36)*	0.082 (2.49)*
*Ln(Crime)*			0.214 (1.65)	0.208 (1.66)
*Ln(Rain)*			−0.431 (2.09)*	−0.433 (2.17)*
*L*n*(Temperature)*			−0.384 (2.65)*	−0.330 (2.58)*
*L(AccessAir)*			0.699 (3.44)**	0.666 (3.41)**
*Ln(NO2)*			0.319 (2.03)*	0.299 (2.01)*
*Ln(CineSeats)*			−0.040 (2.64)*	−0.046 (2.68)*
*Ln(MedAlt)*			−0.060 (1.56)	−0.065 (1.60)
*S*	−0.880 (4.82)**	−0.872 (5.11)**	−0.676 (2.026)*	−0.676 (2.09)*
*W*	−0.367 (2.09)*	−0.366 (2.23)*	−0.357 (−1.61)	−0.342 (1.60)
*E*	−0.453 (2.45)*	−0.397 (2.031)*	0.032 (0.11)	0.086 (0.325)
Year dummies	Yes	No	Yes	No
Obs.	677	677	466	466
Adj. *R*^2^	0.311	0.301	0.321	0.317
LM (Lagrange-Multiplier) test (*p*-value)	58.79 (0.000)	57.91 (0.000)	36.38 (0.000)	36.37 (0.000)
Hausman FE-RE (*p*-value)	122.12 (0.000)	112.19 (0.000)	117.49 (0.000)	110.91 (0.000)
Hausman FE-HT (*p*-value)	7.842 (0.132)	6.635 (0.109)	5.585 (0.232)	2.732 (0.255)

*Notes*: Absolute values of *t*-statistics in parentheses; * significant at 5%; ** significant at 1%. LM test is the Chi-squared of the Breusch-Pagan test comparing the pooling and random-effects estimators. Hausman FE-RE is the Chi-squared of the Hausman test comparing the fixed-effects and random-effects estimator. Hausman FE-HT is the Chi-squared of the Hausman test comparing the fixed effects and Hausman-Taylor estimator. *p*-value is the *p*-value of this test.

**Table 5.** Estimation of the determinants of urban sprawl indices (GLS random effects).

	Dependent variables : Ln(Scatt)			
	(1)	(2)	(3)	(4)
*Constant*	3.168 (5.40)**	3.823 (8.75)**	2.731 (1.39)	3.732 (1.34)
*Ln(POP)*	−0.315 (7.86)**	−0.318 (7.84)**	−0.237 (4.94)**	−0.250 (5.24)**
*Ln(GDPcap)*	−0.124 (2.98)**	−0.188 (10.60)**	−0.035 (1.59)	−0.164 (8.20)**
*Ln(Agriprox)*	−0.141 (4.22)**	−0.144 (4.15)*	−0.067 (1.57)	−0.069 (2.01)*
*Ln(Highway)*	−0.015 (1.51)	−0.019 (1.59)	−0.047 (1.54)	−0.045 (1.72)
*Ln(Crime)*			0.218 (1.68)	0.236 (1.71)
*Ln(Rain)*			−0.208 (1.015)	−0.203 (0.92)
*L*n*(Temperature)*			0.048 (0.083)	0.020 (0.03)
*L(AccessAir)*			−0.153 (0.763)	−0.102 (0.48)
*Ln(NO2)*			−0.422 (2.69)*	−0.401 (2.39)*
*Ln(CineSeats)*			−0.017 (0.16)	−0.014 (0.13)
*Ln(MedAlt)*			0.241 (5.76)**	0.243 (5.44)**
*S*			0.135 (0.40)	0.139 (0.39)
*W*			−0.169 (0.76)	−0.183 (0.77)
*E*			−0.07 (0.27)	−0.15 (0.53)
Year dummies	Yes	No	Yes	No
Obs.	654	654	433	433
Adj. *R*^2^	0.33	0.32	0.39	0.36
LM test (*p*-value)	451.21 (0.000)	453.75 (0.000)	216.09 (0.000)	287.03 (0.000)
Hausman FE-RE (*p*-value)	1.698 (0.782)	1.327 (0.515)	3.326 (0.504)	0.502 (0.777)

*Notes*: Absolute values of *t*-statistics in parentheses; * significant at 5%; ** significant at 1%. LM test is the Chi-squared of the Breusch-Pagan test comparing the pooled and random-effects estimators. Hausman FE-RE is the Chi-squared of the Hausman test comparing the fixed-effects and random-effects estimators. *p*-value is the *p*-value of tests.

Some qualifications need to be made regarding the use of the Hausman-Taylor estimator, in the case of the *ArtifArea* index. Although the fixed-effects estimator is not an option in our study, since it does not allow the estimation of the coefficients of the time-invariant regressors, it is still useful in order to test the strict exogeneity of the regressors that are used as instruments in the Hausman-Taylor estimation. Thus, when strict exogeneity for a set of regressors is rejected, others must be considered to act as instruments in the estimation. Once the second Hausman test has identified which regressors are strictly exogenous, they are subsequently used as instruments in the Hausman-Taylor estimation.

After testing several configurations, we retain *POP* as endogenous, while *GDPcap* and all time-invariant variables are exogenous. Only this configuration allowed us to obtain estimates close to the fixed-effects for time-varying variables. In addition, the Hausman test confirms the consistency of the Hausman-Taylor estimator (see bottom of Table 4). In addition to this statistical procedure, the exogeneity of GDP per capita can be intuitively explained by the fragmented nature of LUZs, where the agglomeration effect could be much less relevant. This would especially hold in the case of strongly fragmented urban areas. Certain costs related to fragmented urban areas (e.g. infrastructure costs and productivity losses due to additional time spent on commuting) may adversely affect the economic outcome.

Table 4 reports the results of a regression for the *ArtifArea* index obtained using the Hausman–Taylor estimator. Two configurations of the model are presented. The first includes only the main variables of the monocentric city model, i.e. population, GDP per capita, agricultural rent proxy and transportation costs proxy (columns (1) and (2)). The second configuration adds all of the explanatory variables selected in this study (columns (3) and (4)). Furthermore, year and regional dummies are used to control for time-specific changes in the sprawl indices caused by other factors.

All the coefficients of the main independent variables emerge as significant with the expected signs (columns (1) and (2)). The Population coefficient is significant and positive, ranging between 0.288 and 0.329. The GDP per capita coefficient is also significant with a positive sign, varying between 0.168 and 0.281. The sign on the coefficient of Agriprox, the proxy for agricultural land values, is negative as predicted by the monocentric model. The higher the agricultural land value, the slower the expansion of artificial area. The coefficient on the transportation cost proxy (Highway) is positive which is also as expected. When transportation networks are dense, the cost of travel is low and the artificial area is relatively large. As other explanatory variables are added, the main variables of the monocentric model remain significant with the expected signs. This is still true with or without the dummies for years.

Interestingly, this study highlights the importance of agricultural productivity in limiting the expansion of urban areas. Unlike previous studies, a relatively high coefficient is observed for the agricultural rent proxy, ranging from −0.265 to −0.279. This means that agricultural productivity can be a genuine barrier to urban sprawl in Europe. This reflects the fact that in Europe, agriculture at the urban fringe is often highly intensive, offering relatively high yields and profits.

The climatic variables (*Rain* and *Temperature*) have a significant and negative effect, which reflects the tendency towards urban sprawl in temperate climates. The connectivity of cities to the rest of the world, measured through the relative importance of the nearest airport (*AccessAir*), is also significant and positive. Generally, cities with a major airport attract significant economic activity and therefore expand. The coefficient of the variable *NO2* is significant and positive. Thus, pollution recorded in the central city tends to encourage households to move to suburban areas, promoting sprawl. The cultural attractiveness of the city, approximated by the number of cinema seats, is significant and negative, suggesting that attractive cultural amenities in the centre of the urban area discourage outward sprawl that makes those amenities less accessible. The coefficients of the variables *Crime* and *MedAlt* are not significant, but show the expected signs. A high crime rate in the central city promotes urban expansion, encouraging households to settle in suburban areas. As expected, increasing altitude acts as a brake to the expansion of cities.

Returning to the *Scatt* index, where the Hausman test rejects the fixed-effects estimator in favour of the random-effects model. Table 5 reports the results of the regression and various statistical tests. Again, two configurations, with and without *year dummies*, are considered. Columns (1) and (2) includes the main variables of the monocentric city model, while Columns (3) and (4) add the other explanatory variables.

The results reported in columns (1) to (4) in Table 5 are consistent. The low adjusted *R*^2^ values and non-significance of several variables, show that fragmentation is not necessarily influenced by the same set of variables that determines spatial scale. In all cases, coefficients for *POP* and *GDPcap* are negative and significant suggesting that larger populations and higher income levels in an urban area may be linked to lower rates of fragmentation. Therefore increases in population and per capita income are associated with cities that are both larger and more compact. This reflects the strong demand for land in more affluent LUZs and the associated levels of population growth. Such demand may lead to a reduction in the number of urban fragments, as discrete settlements start to expand and merge with each other, or with the central city. Such phenomena can be influenced by urban planning policies, which may be designed to encourage development within these interstitial spaces rather than around the fringes of the LUZ.

Furthermore, the coefficient of the agricultural land value proxy is also negative but not always significant. As might be expected, high agricultural land productivity should constrain urban fragmentation by limiting the amount of land available for development. The opposite might be expected for less productive land provided that other factors (e.g. topography or drainage) are favourable to development. The results reported here, suggest some level of heterogeneity in the agricultural activities within each LUZ, resulting in complex land-use patterns specific to each area. The transport cost proxy also had a negative coefficient, and again this was not always significant. Of the other explanatory variables, only *NO2* and *MedAlt* were significant in the models. The pollution proxy has a negative impact on scattering, reflecting a tendency towards greater fragmentation in cities experiencing higher levels of air pollution. However, the effect of altitude is positive; thus cities located in urban areas at higher altitudes are likely to be more fragmented, possibly as a result of the local terrain.

### Investigating the effects of variables that vary over time

The relative importance of variables that vary over time (*POP* and *GDPcap*) in explaining changes in the sprawl index, can be ranked according to the magnitudes of their elasticities. However, this criterion can be misleading, because the total effect of one factor on another over time, depends on both the magnitude of the elasticity and the change in the variable.

Decomposition analysis was used to help understand the effect of these time-varying variables on the dependent variables. This approach accounts for both the size of the marginal effects and the magnitude of the change in the explanatory variables. Table 6 reports the results of the decomposition analysis for both *ArtifArea* and *Scatt* index.^13^*GDPcap* is the most important factor affecting change in artificial area. Over 77% of the growth of urban areas between 1990 and 2006 is explained by increases in income per capita. However, population growth explains only 4.96% of urban area growth. Other explanatory variables explain another 17.44% of variation in the expansion of urban areas. By contrast, 14% of the decline in scattering is explained by population growth and 26.24% by growth in income per capita.

**Table 6.** Decomposition analysis of sources of urban sprawl indices.^a^

Variables		*Ln(ArtifArea)*			*Ln(Scatt)*		
	(1)	(2)	(3)	(4)	(5)	(6)	(7)
	Changes in variables (percent)	Estimated parameter	Impact on ArtifArea	Contribution (percent)	Estimated parameter	Impact on Scatt	Contribution (percent)
*Ln(POP)*	5.36	0.170	0.911	4.96	−0.237	−1.27	14.00
*Ln(GDPcap)*	68.00	0.210	14.28	77.60	−0.035	−2.38	26.24
Residual				17.44			59.76
*ArtifArea*	18.40			100			
*Scatt*	−9.07						100

*Notes*: ^a^The decomposition analysis follows three steps. First, the percentage change of each variable between 1990 and 2000 is calculated (column 1). Then column 1 is multiplied by parameters estimated for each index (columns 2 and 5) to obtain the impact of each time-varying variable on both indices, respectively (columns 3 and 6). Finally, the impact of each variable is divided by the percentage change in *ArtifArea* (18.4%) and Scatt (−9.07%) to obtain the contribution of each variable to changes in *ArtifArea* (column 4) and *Scatt* (column 7).

The significance of this decomposition analysis is twofold. First, income growth is shown to be by far the most important cause of urban expansion. Second, other factors are found to be more important than changes in income and population in explaining the fragmentation of urban areas within LUZs.

## Conclusions

Using the framework of the monocentric city model, this paper has empirically investigated the determinants that influence urban sprawl across a large set of European cities. The phenomenon of sprawl was examined both as an increase in the spatial scale of urban areas and as a process of fragmentation, where urban area is shown to be characterised by a number of discrete parcels of urban settlement scattered around the central city. For each city in our sample, data on these two dimensions of urban sprawl were accurately measured using GIS software. Based on the urban economic literature on urban sprawl, a set of potential explanatory variables was drawn up and appropriate data collected from a range of existing sources (e.g. Eurostat, Urban Audit, ESPON). Where data on potential explanatory variables were not available, a suitable proxy variable was constructed.

Data were obtained for these variables over three reference years (1990, 2000 and 2006). The use of panel data allows unobservable individual heterogeneity to be controlled but also means that a simple OLS estimator is unlikely to be suitable, as this would not account for such unobservable heterogeneity across cities. Several different estimators were considered and statistical tests were performed to determine the ability of each to account for the specific structure of the panel data for the two aspects of sprawl measured by the study. The Hausman-Taylor estimator was used in the case where sprawl is measured in terms of changes to the urban (artificial) area, but where the dependent variable is an index of fragmentation (i.e. scattering) a random-effects estimator was adopted.

Our results are robust and when urban sprawl is approximated by the spatial scale, i.e. changes to the artificial area within the LUZ, they clearly confirm the predictions of the monocentric city model. Thus, the coefficients of the main explanatory variables in the model are significant, with the expected signs. In addition, the significance of these variables does not change when other explanatory variables are introduced. While increasing income per capita and population growth are clearly associated with the expansion of urban areas, the models reported in this paper also suggest a possible link between the productivity of adjacent agricultural land and a more restricted outward growth of cities. High productivity maintains or increases land values and makes development on the urban fringe more expensive and therefore less attractive. This economic restriction to the supply of available land may be supported by planning regulations, which limit the availability of land in the urban fringe for development, while the value of agricultural land close to urban areas might be increased owing to proximity to markets for some high-value products such as soft fruits and salad vegetables.

There is less obvious correlation between the fragmentation of urban areas and the growth of income and population. Other factors, such as altitude or terrain, are shown in the model to be associated with fragmentation but much of the variation is left unexplained. It is suggested that urban planning policies and land availability may be particularly influential in determining the level of fragmentation, along with any other factors that reduce the outward growth of cities and therefore encourage in-fill development in the interstices between fragments.

Some limitations to this study must be acknowledged, such as our current inability to include variables relating to important political and institutional factors (e.g. land supply and zoning), that are likely to affect both urban scale and fragmentation. The model also omits information on some specific geographical features therefore limiting our ability to explore the variation in urban sprawl indices more deeply. It is also possible that there may be complex interactions between some environmental factors (such as coastal and mountain amenities) and urban sprawl, that are not accounted for in our model.

Although we have not accounted explicitly for the role of land-use policies (mainly because of the lack of data), our study may provide some possible insights into the design of policies seeking to control sprawl. While environmental and landscape protection are important aims, such policies should not ignore the important economic mechanisms that can drive urban sprawl. This research suggests that in many cities, urban sprawl may be associated with increasing wealth. Therefore policies that limit the expansion of urban areas may risk restricting economic growth, as house prices within the LUZ increase, development land becomes scarce and individuals and businesses decide to relocate to other cities where there is still room for new development on the periphery.

Policy makers reluctant to place regulatory restrictions on sprawl but who are concerned about the loss of environmental quality or amenity from the development of the urban fringe, may wish to consider other policies that use the market to discourage the outward expansion of cities. Our results suggest that agricultural productivity, and by extension profits, can restrict development by driving up land prices around cities. Therefore the adoption of policies that have a positive impact on farm incomes on the urban periphery could have a direct effect on reducing the likelihood of outward sprawl, while at the same time potentially encouraging the development of non-urban areas within the LUZ boundary, thus reducing urban fragmentation and making the city more compact.

## Notes

1. Patacchini and Zenou (2009) used data provided by the Urban Audit database.

2. The UA database arises from a project coordinated by Eurostat that aims to provide a wide range of indicators of socio-economic and environmental issues. These indicators are measured across four periods: 1989–1993, 1994–1998, 1999–2002, 2003–2006. For further details, refer to : http://epp.eurostat.ec.europa.eu/portal/page/portal/region_cities/city_urban.

3. For further details, refer to: http://eea.europa.eu/.

4. The supra-national regions are defined as follows: Southern European cities (Cyprus, Portugal, Spain, Italy, Malta, Slovenia), Western European cities (Austria, Germany, Belgium, France, Ireland, Luxembourg, Netherlands, UK). Northern European cities (Denmark, Sweden, Finland, Estonia, Latvia, Lithuania) and Eastern European cities (Poland, Romania, Slovakia, Hungary, Czech Republic, Bulgaria).

5. ESPON is a European research programme, which provides pan-European evidence and knowledge about European territorial structures, trends, perspectives and policy impacts which enables comparisons amongst regions and cities. For further details, see: http://database.espon.eu/.

6. Purchasing Power Standards (PPS) reflect price ratios between countries and are expressed in a single currency. They thus eliminate from GDP both differences in currency expression and in price levels between countries.

7. Total population includes all individuals who reside within the LUZ.

8. As there is a strong correlation between ArtifArea and density, similar results are obtained for both dependent variables, except for the sign of some coefficients.

9. Our panel data is unbalanced because of randomly missing observations. We used ANOVA to estimate the error component from the unbalanced random effect term. Unsurprisingly, as our model is linear, the error term has no significant effect on the system.

10. The Lagrange-Multiplier test (LM test) is used to test for the existence of individual heterogeneity, i.e. testing whether or not the pooled OLS is an appropriate model (Breusch and Pagan, 1980). The OLS hypothesis is unsurprisingly rejected for all configurations. The results of the LM test are reported at the bottom of Tables 4 and 5.

11. For more details see Hausman and Taylor (1981), Wooldridge (2002) and Baltagi et al. (2003).

12. All estimates presented in this paper are obtained using the Plm package of R. For details see Croissant and Millo (2008). Fixed effects estimates are not reported in the paper, but are available on request from the authors.

13. Estimated parameters for *ArtifArea* correspond to those obtained in Table 4 column 3. Estimated parameters for *Scatt* correspond to those reported in Table 5 column 3. Considering estimated parameters without *year dummies* does not change the conclusions drawn from the decomposition analysis.
